# Prevalence of Internet Addiction and Its Associated Factors Among Undergraduate Students at the University of Botswana: A Cross-Sectional Study

**DOI:** 10.1177/00469580261432826

**Published:** 2026-03-24

**Authors:** Lorato Keutule, Keneilwe Molebatsi, Anthony A. Olashore

**Affiliations:** 1 Department of Psychiatry, Faculty of Medicine, University of Botswana, Gaborone, Botswana

**Keywords:** internet addiction, depression, anxiety, university students, mental health, Botswana

## Abstract

Internet Addiction (IA) is an emerging public health concern, especially among young adults. It has been associated with various psychiatric conditions, including depression and anxiety. Despite increasing internet access in Botswana, local data on IA and its psychological correlates remain limited. This study investigates the prevalence and patterns of internet addiction and its association with depression and anxiety among undergraduate students at the University of Botswana. A cross-sectional study was conducted among 500 undergraduate students from 5 randomly selected faculties. Participants completed the Young Internet Addiction Test (YIAT), Patient Health Questionnaire-9 (PHQ-9), and Zung Self-Rating Anxiety Scale (SAS). Data were analyzed using descriptive statistics and multiple linear regression. The mean age was 21.42 years (SD = 4.36); 52.2% were female. The prevalence of IA was 88.4%. Social networking was the most common reason for internet use (91.8%). High depression scores were observed in 64% of participants, and moderate anxiety levels in 47%. Depression (*B* = 0.26; *P* < .001), anxiety (*B* = 0.12; *P* < .02), and time spent online (*B* = 0.33; *P* < .001) were significant predictors of IA. IA is common among university students in Botswana and is linked to depression and anxiety. These findings underscore the importance of routine screening, education, and psychological support in universities.

## Introduction

The internet has become an indispensable tool in modern life, revolutionizing how people communicate, access information, work, and socialize. Its benefits are numerous, particularly in education and business, where connectivity enhances productivity and learning.^1^ However, as digital engagement becomes increasingly embedded in daily life, a new class of behavioral challenges has emerged, among them Internet Addiction, a phenomenon characterized by excessive, compulsive internet use that interferes with daily functioning, emotional well-being, and interpersonal relationships.^2^

Though not formally recognized in the DSM-5 as a distinct disorder, IA shares many core features with impulse control and substance use disorders. These include tolerance, withdrawal, loss of control, and continued use despite negative consequences.^2^ Some scholars propose that IA should be conceptualized within the broader framework of behavioral addictions, alongside gambling and gaming disorders.^3^ It may manifest through compulsive behaviors related to online gaming, social networking, pornography, gambling, shopping, and information seeking.^3^ Common across these activities is the engagement of the dopaminergic reward system, which reinforces online behavior and fosters dependency patterns similar to those seen in substance use.

Emerging neuroimaging and neuropsychological evidence suggest that IA involves dysregulation in the brain’s reward, executive control, and emotional regulation systems, much like substance use disorders.^4,5^ These findings support the conceptualization of IA as a disorder of behavioral reinforcement and impaired inhibitory control, similar in neural basis to traditional addictions.

University students represent a particularly vulnerable group for IA, because they are young, technologically adept, and under substantial academic, emotional, and social pressure. Their heavy reliance on the internet for education, entertainment, and social interaction, combined with high autonomy and limited external monitoring, places them at increased risk for compulsive online behavior. Research has consistently demonstrated elevated IA rates in university settings.^6^ A study in Taiwan estimated IA prevalence among students to be 33.9% while a Nigerian study found rates as high as 78.6%.^7^ These figures reflect a global trend of increasing IA among youth.

Crucially, IA has been associated with a host of mental health consequences, most notably depression and anxiety.^8^ Studies have reported strong associations between IA and elevated levels of psychological distress, with some suggesting a bidirectional relationship.^8,9^ Individuals with preexisting mental health conditions may turn to the internet as a coping mechanism to escape emotional discomfort, loneliness, or low self-esteem. Conversely, excessive internet use can exacerbate psychiatric symptoms by reducing sleep quality, impairing academic performance, and diminishing real-world social engagement. It has also been shown that IA correlates with poor sleep hygiene, increased suicide ideation, social anxiety, substance use, and overall decreased quality of life.^10^

In Africa, despite the exponential growth of internet access, especially in urban centers, relatively few studies addressed IA. Those that do exist report alarmingly high prevalence rates; for instance, an Ethiopian study reported a prevalence of over 43% among university students,^11^ while a Tunisian^12^ and a Nigerian^13^ university-based study reported prevalence rates of 54% and 78.6%, respectively.

In Botswana internet usage has expanded rapidly over the past decade, driven by growing mobile penetration, affordable data packages, and investments in national digital infrastructure. According to recent statistics, internet penetration in Botswana reached approximately 77% in early 2024, up from just 61% in 2022. Anecdotal evidence and qualitative observations suggest that many students spend several hours online daily, often engaging in non-academic activities like social networking and streaming. This raises concerns about potential neglect of academic responsibilities, disrupted sleep patterns, and declining mental well-being. Despite this surge in connectivity, little is known about how digital behavior is impacting the mental health of youth in Botswana.

Although we expect IA to be common among our cohort and linked to psychological disorders, there is no current data to support this. Therefore, we aimed to assess its prevalence and related factors among University of Botswana students. We believe this will raise awareness among management about the seriousness of these issues and serve as a basis for future research, intervention strategies, and policy development.

## Methods

### Study Design and Setting

A cross-sectional design was used for the present study. The setting was the University of Botswana, Gaborone campus, the country’s premier tertiary institution, known for its diverse and representative student population. The institution comprises 8 faculties that span various academic disciplines and draws students from different socioeconomic, cultural, and geographic backgrounds, making it ideal for behavioral research involving youth and digital engagement.

### Study Population and Inclusion Criteria

The study targeted undergraduate students in Years 2, 3, and 4, as they had adequate university experience and digital exposure necessary for the objectives of the study. First-year students were excluded due to limited campus experience and ongoing registration during data collection. Other exclusion criteria included students who were unable to give informed consent, such as those below the legal age.

### Sample Size Determination

To assess internet addiction and its associated factors among undergraduate students, the minimum sample size required was calculated using Cochran’s formula for determining sample sizes in cross-sectional studies^14^:

$$N=\frac{p(1-P)\;.\;z^{2}}{d^{2}}$$

Where: *N* = minimum sample size required, *p* = prevalence of internet addiction (31%) in a lower-middle-income country like Botswana, *z* = 1.96 (*Z*-value corresponding to a 95% confidence level), *d* = desired level of precision (5% or 0.05). By plugging the values into the formula:

$$N=\frac{0.31\left(1-0.31\right).1.96^{2}}{0.05^{2}}=329\;\mathrm{participants}$$

Thus, the minimum sample size required to assess the relationship between internet addiction and mental health symptoms (anxiety and depression) was 329 participants. In anticipation of potential non-response or loss to follow-up, we applied an adjustment factor based on a similar study conducted in Tanzania. This is calculated using the following formula:

$$N_{2}=\frac{n_{1}}{1-LFT}$$

Where: *n*_1_ = Initial sample size (329), LFT = loss to follow-up rate (10% or 0.1).

Applying this, the adjusted sample size becomes:

$$N_{2}=\frac{329}{1-0.1}=365\;\mathrm{participant}$$

Thus, the adjusted minimum sample size required to account for non-responses was 365 participants. To ensure the study’s robustness, the final sample size was rounded up to 370 participants. However, a total of 500 participants ultimately participated in the study, surpassing the minimum required sample size, which helps to further enhance the study’s statistical power. A limitation of this approach is that the total number of eligible students was not recorded during data collection. As a result, a formal response rate could not be calculated. This constrains the assessment of potential non-response bias and limits the evaluation of the sample’s representativeness.

### Sampling Technique

A multistage stratified random sampling method was employed due to its efficiency, representativeness, and cost-effectiveness. The process included:

1. Stratification by Faculty: The entire student population was divided into 8 faculties.
2. Random Faculty Selection: Five faculties were selected using the paper ballot (lottery) method.
3. Random Department Selection: Two departments were randomly chosen from each of the 5 faculties.
4. Class Selection: Three classes per selected department were then chosen at random.
5. Participant Selection: Within the selected classes, students were randomly sampled using a ballot method. This approach ensured diversity across academic disciplines and reduced selection bias.

### Recruitment and Procedure

Two research assistants were trained in ethical conduct, engagement strategies, and proper questionnaire administration. The data collection took place over 10 weeks (August 12-October 18, 2024), structured to minimize classroom disruption. Coordination involved contacting lecturers, securing permission, and scheduling suitable times during lectures.

On data collection days, the research assistants arrived early, introduced the study, and emphasized the voluntary nature of participation. The paper ballot method was used to select participants. Each consenting student received 4 self-administered questionnaires. Participants completed the surveys independently in about 20 min, in either English or Setswana, according to preference. Assistants were present to answer questions but avoided influencing responses.

### Ethical Considerations

The study received ethical clearance from the University of Botswana research and Ethical Review Committee. Key ethical protocols included informed consent obtained in writing before participation, assurance of voluntary participation with the right to withdraw at any point, anonymity and confidentiality maintained through secure storage and restricted data access and the minimization of harm, with a psychiatric resident available for support, and referrals to counseling or psychiatric services for distressed participants.

### Measurement Instruments

Four validated self-administered tools were used:

1. Socio-Demographic Questionnaire: Captured participant details including age, gender, academic program, relationship status, religious affiliation, place of residence, income, internet access habits, and screen time.
2. Young’s Internet Addiction Scale (YIAS): A 20-item tool measuring internet addiction severity based on 6 domains: salience, withdrawal, tolerance, conflict, craving, and mood modification. Items were rated on a 5-point Likert scale. The tool has a Cronbach’s alpha of .84, indicating high reliability. The cut-off points used for classifying Internet Addiction (IA) based on Young’s Internet Addiction Test (YIAT). The following scoring criteria were applied: Normal use: 0 to 30, Mild IA: 31 to 49, Moderate IA: 50 to 79, Severe IA: 80 to 100.^15^
3. Patient Health Questionnaire-9 (PHQ-9): A 9-item scale used to assess the severity of depression symptoms, widely used in primary care and mental health settings. It provides a score ranging from 0 to 27.^16^ In the current study a cut off of 10 was used.
4. Zung Anxiety Scale (SAS): A 20-item tool assessing psychological and somatic symptoms of anxiety. Each item is rated from 1 (“a little of the time”) to 4 (“most of the time”), with some reverse-coded questions to prevent response bias. The internal consistency is satisfactory, with a Cronbach’s alpha of .82.

### Data Analysis

Data were entered into Microsoft Excel, cleaned, and exported to SPSS version 25 for statistical analysis. No data was missing from the data that was collected. Both descriptive and inferential statistics were used. Continuous variables (eg, age, screen time) were analyzed using means and standard deviations. Categorical variables (eg, gender, relationship status) were described using frequencies and percentages. Internet usage patterns were visually presented using graphs and tables.

Since the sample size was sufficiently large, parametric statistics were deemed appropriate, regardless of the normality of the outcome variables.^17^ The literature was used to inform the selection of variables for building the regression model; a single-step multiple linear regression model was used to identify predictors of internet addiction. The level of statistical significance was set at *P* < .05. The *P*-*P* plot displayed a normal distribution with points forming a reasonably straight diagonal line, indicating no significant departure from normality.^18^

## Results

A total of 500 undergraduate students participated in the study. The mean age of participants was 21.42 years (SD 4.36), with a range from 18 to 26 years. Females comprised 52.2% of the sample (Table 1). More than half of participants (64.8%) had high depression scores, while anxiety levels varied, with 56% having normal anxiety levels, 34% experiencing mild anxiety, 9.4% reporting moderate anxiety, and 0.6% suffering from severe anxiety. Substance use was reported by 32% of the participants.

**Table 1.** Socio-demographic Characteristics of the Participants.

Variable	Statistics	
Age (SD)	21.42 ± 4.36 years	
	N	Percentage
Gender	500	100
Male	239	47.8
Female	261	52.2
Relationship status		
Single	316	63.2
Married/in a relationship	184	36.8
Religion	500	100
Christianity	470	94
Others	30	6
Residence	500	100
On campus	42	8.4
Off campus	458	91.6
Faculty of study	500	100
Business	112	22.4
Social sciences	70	14
Science	80	16
Humanities	114	22.8
Education	124	24.8
Year of study	500	100
Second	173	34.6
Third	194	38.8
Fourth	133	26.6

**Figure 1.**
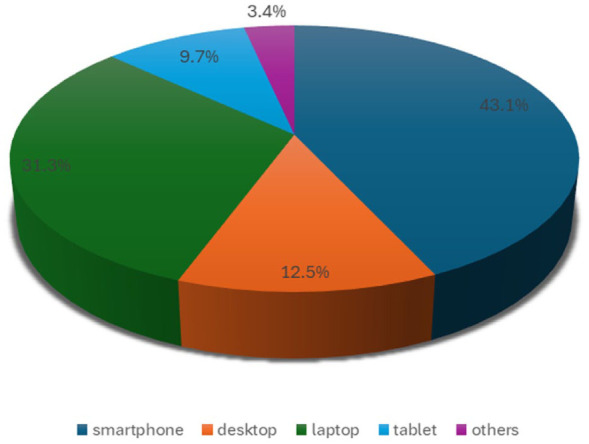
Mode of internet access.

**Figure 2.**
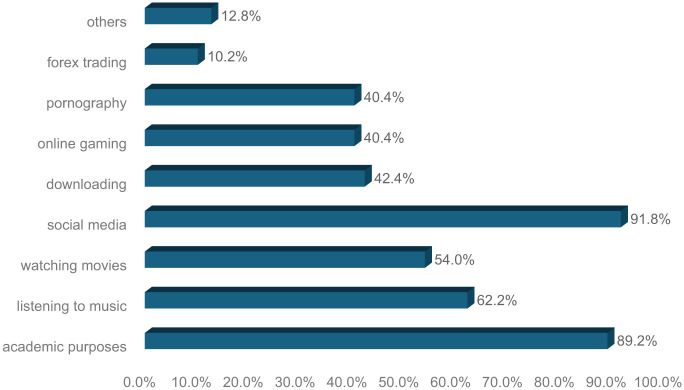
The primary purpose of Internet use among participants.

The majority of participants (43.1%) accessed the internet using smartphones, making it the most commonly used device. Laptops were the second most frequently used device (31.3%), followed by desktop computers (12.5%). Tablets were used by 9.7% of the respondents, while a small proportion (3.4%) relied on other devices such as smart TVs and smartwatches (Figure 1). Social media emerged as the most common reason for internet use among participants, with 91.8% indicating that they primarily used the internet for social networking. Academic purposes were also a major reason for internet use, reported by 89.2% of participants. Other common internet activities included listening to music (62.2%), watching movies (54%), downloading content (42.4%), and online gaming (40.4%). A smaller percentage of students used the internet for activities such as pornography (40.4%), forex trading (10.2%), and other miscellaneous purposes (12.8%; Figure 2).

**Figure 3.**
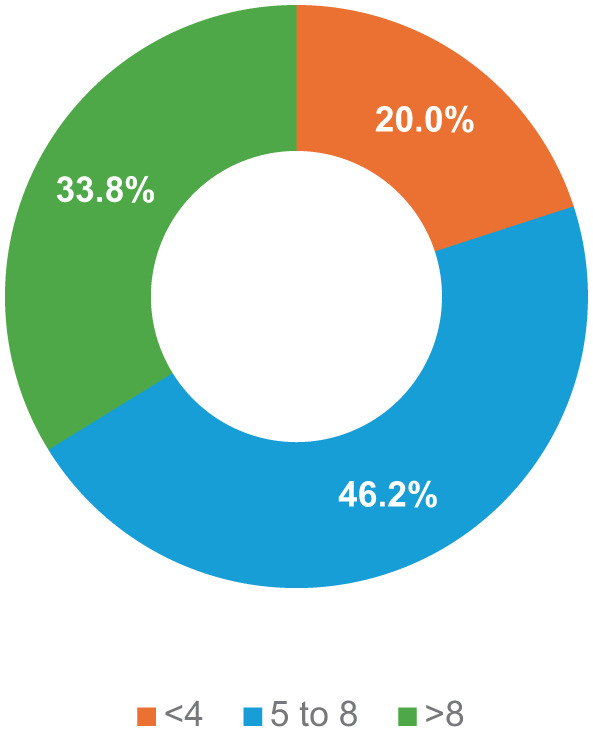
Time (hours) spent using the Internet/screen time per day among participants.

Regarding screen time, the largest proportion of participants (46.2%) reported spending between 5 and 8 h per day on the Internet. About one-third (33.8%) used the internet for less than 4 hours daily, while 20% reported spending more than 8 hours online per day (Figure 3).

### Prevalence of Internet Addiction

**Figure 4.**
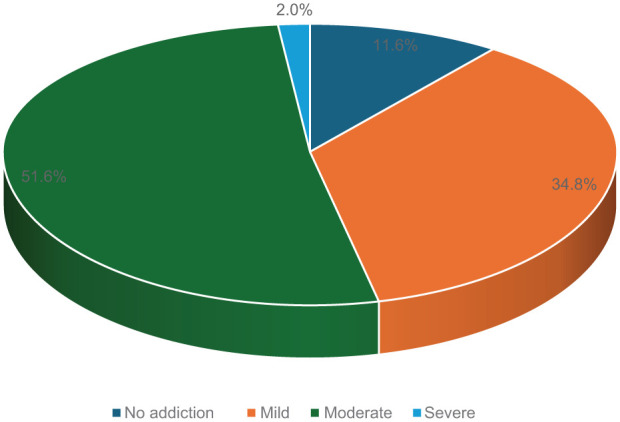
Internet addiction categories among the participants.

The prevalence of internet addiction among the participants was found to be high, with 88.4% exhibiting some level of addiction. Among these, the majority (51.6%) had moderate internet addiction, while 34.8% had mild addiction. Severe internet addiction was observed in 2% of the participants, whereas only 11.6% showed no signs of internet addiction (Figure 4).

### Multiple Linear Regression Predicting Internet Addiction

The multiple linear regression analysis revealed that depression (*B* = 0.26; *P* < .001), anxiety (*B* = 0.12; *P* = .02), and the number of hours spent on the internet (*B* = 0.33; *P* < .001) were significantly associated with IA, and the total variance explained by the model was 30%, *F* (7, 492) = 29.6, *P* < .01 (Table 2).

**Table 2.** Multiple Linear Regression Showing Predictors of Internet Addiction.

Variables	Beta	*t*	*P*-value
Sex/gender	0.03	0.74	.46
Age	0.01	0.23	.82
PHQ total	0.26	4.74	**.00**
SAS total	0.12	2.29	**.02**
Total OSLO	0.02	0.52	.61
Substance use	−0.06	−1.42	.16
Hours on the internet	0.33	8.36	**.00**

*Note*. Significant values are in bold.

## Discussion

We set out to determine the prevalence and correlations of IA among undergraduate students at the University of Botswana, with a particular focus on its relationship with depression and anxiety. The results revealed a strikingly high prevalence of IA, with 88.4% of students meeting criteria for mild, moderate and severe addiction. It is important to note that YIAT assesses a spectrum of problematic internet use rather than providing a clinical diagnosis of addiction. Consequently, the high prevalence observed in this study likely reflects widespread at-risk or maladaptive internet use rather than severe addictive pathology among most students. This unusually high prevalence may reflect growing dependence on digital platforms for academic tasks, social communication, and entertainment, combined with widespread smartphone penetration, campus-wide Wi-Fi access, and increased digital engagement following the COVID-19 pandemic. Furthermore, IA was significantly predicted by daily internet use, depression, and anxiety scores, supporting the role of psychological vulnerability in problematic online behavior and aligning with findings from Fallah et al,^19^ who emphasized that impaired emotion regulation and deficits in executive functioning can increase susceptibility to internet addiction.

The present study found that smartphones were the dominant mode of internet access among students, consistent with findings by Kheradmand et al^20^ and Tateno et al.^21^ However, while smartphone use was widespread, we did not observe any independent association between smartphone access and IA. This suggests that device type alone may not predict addiction; rather, behavioral and psychological factors appear more influential in driving compulsive use.^22^ This interpretation is consistent with recent literature emphasizing the importance of emotional regulation difficulties, impulsivity, and reward sensitivity-mechanisms.^19^

A key finding that warrants deeper interpretation is that substance use, though reported by 32% of participants, did not emerge as a significant predictor of IA in the multivariable model despite showing bivariate associations. One possible explanation is the non-standardized and broad nature of the substance use questions used in this study, which may have limited measurement precision compared with validated tools used in prior research.^23^ Another explanation is that stronger predictors such as depression and anxiety, known to have substantial overlap with IA, may have attenuated the independent contribution of substance use once included in the regression model. This pattern aligns with Fallah et al,^19^ who argue that emotional and cognitive vulnerabilities may mediate addictive behaviors more strongly than external behavioral comorbidities. Nonetheless, the potential for co-occurrence between IA and substance use underscores the need for improved screening and future studies using standardized measures.

In the present study, the majority of students (51%) exhibited moderate IA, while 2% were severely addicted according to YIAT scoring. Differences in sociocultural expectations, online behavior norms, and internet accessibility may help explain the variability. Furthermore, the absence of standardized diagnostic criteria for IA, reflected in its exclusion from DSM-5 and ICD-10, continues to challenge cross-study comparisons. Despite these limitations, our findings highlight the need for universities to implement preventive interventions tailored to students’ digital behavior patterns.

More than 90% of participants reported primarily using the internet for non-academic purposes, particularly social media engagement. This aligns with previous findings from the University of Botswana^24^ and international studies by Saikia et al^1^ and Bisen et al,^25^ which documented social networking as the dominant online activity among university students. Social media engagement is associated with social influences, such as peer pressure and the fear of missing out (FOMO), which may contribute to more frequent online interaction.^22,26^ These patterns reflect possible mechanisms but should be interpreted as associations, not causal relationships, given the cross-sectional study design. High levels of recreational use raise concerns about negative outcomes such as reduced academic performance, psychological distress, cyberbullying exposure, and sleep disruption. These patterns are consistent with frameworks describing IA as a maladaptive coping mechanism for underlying emotional difficulties where impaired emotion regulation increases vulnerability to compulsive digital use.^19^

In comparing our findings with previous literature, the prevalence identified in this study is comparable to reports from Ethiopia and India, both of which used the Young Internet Addiction Test (YIAT) and found similarly high levels of problematic internet use.^1,27^ However, our prevalence is notably higher than that reported in developed countries such as the UK (3.2%) and Lebanon (4.1–8.3%).^28,29^ These differences may be due to variations in cutoff scores, cultural norms regarding internet use, stages of digital maturity, and methodological inconsistencies across studies. For example, our use of a YIAT cutoff score of 45 compared with a higher threshold of 50 in Younes et al^30^ may have contributed to a larger proportion of students being classified as addicted. The psychological profile of students in our setting may mirror the pattern described by Fallah et al^19^ where emotional dysregulation plays a mediating role in escalating internet use from habitual to addictive.

Our study identified total time spent online as a significant predictor of IA, a finding consistent with research by Kutty et al^31^ in Malaysia and Acharya et al^32^ in Nepal, both of which reported higher IA risk among individuals using the internet for more than four to five hours daily. These studies attribute excessive use to diminished self-control, a key feature of IA. However, the link between duration and addiction remains contested. Some scholars argue that content type, such as online gaming, social media, or gambling, may be more critical than the sheer number of hours spent online.^32^ This perspective suggests that compulsive behavior patterns, rather than time alone, drive addiction. Given these insights, screen time management strategies are recommended. These may include integrating screen time trackers, timers, and automated alerts into smartphones and laptops to increase user awareness. Such tools could also deliver periodic warnings about the negative effects of excessive screen time on mental health, social relationships, and daily functioning. Findings from the present study indicate a link between IA and psychological disorders such as depression and anxiety. This is consistent with the study by Saikia et al,^1^ which found a link between IA and both depression and anxiety,1 as well as the research conducted in Malaysia, which reported a similar finding.^33,34^

Our study also confirmed significant associations between IA and psychological disorders such as depression and anxiety, supporting findings from Saikia et al^1^ and studies from Malaysia.^22^ This relationship is considered bi-directional, where each condition may contribute to and worsen the other.^8,9,35^ Excessive internet use may lead to social withdrawal, loneliness, and sleep disturbances, while increased use of social media exposes individuals to negative social comparisons and cyberbullying all factors linked to psychological distress.^36^ Conversely, individuals with depressive or anxious symptoms may be more likely to engage in excessive online activities as a maladaptive emotion-regulation strategy, a mechanism described in the literature and specifically emphasized by Fallah et al^19^ While these excessive online activities offer temporary relief, they actually reinforce compulsive use and contribute to worsening mental health.^35,36^ This cycle underscores the importance of integrated mental health interventions addressing both IA and psychological symptoms.

### Strengths and Limitations of the Study

This study has several limitations, the cross-sectional design restricts causal interpretation, and self-report instruments are subject to social desirability and recall bias. Cultural stigma surrounding mental health may have influenced responses on the PHQ-9 and SAS. Additionally, the absence of clinical diagnostic interviews limits confirmation of depression and anxiety. The study was conducted at a single university, which may reduce generalizability. Furthermore, the YIAT cutoff scores used have not been validated within the cultural context of Botswana, potentially affecting classification accuracy. Despite these limitations, the study is the first to document IA and its correlates among undergraduate students in Botswana, providing valuable data for future policy and intervention planning.

## Conclusion and Recommendations

The study found an alarmingly high prevalence of IA among undergraduate students at the University of Botswana, with 88.4% of participants exhibiting mild to severe symptoms of addiction. The findings demonstrate a clear and significant relationship between IA, daily internet use, depression, and anxiety. These results reflect the increasing psychological burden of digital overuse among young adults in academic settings, particularly in rapidly digitizing contexts such as Botswana. The widespread use of the internet for purposes other than its intended functions, particularly for social media, gaming, and pornography, should prompt management to regularly update the server. This includes periodic monitoring, identifying potentially harmful and inappropriate content, and taking steps to block it. Also, IA should be recognized as a potential mental health challenge among undergraduate students.

## Supplemental Material

sj-docx-1-inq-10.1177_00469580261432826 – Supplemental material for Prevalence of Internet Addiction and Its Associated Factors Among Undergraduate Students at the University of Botswana: A Cross-Sectional Study Supplemental material, sj-docx-1-inq-10.1177_00469580261432826 for Prevalence of Internet Addiction and Its Associated Factors Among Undergraduate Students at the University of Botswana: A Cross-Sectional Study by Lorato Keutule, Keneilwe Molebatsi and Anthony A. Olashore in INQUIRY: The Journal of Health Care Organization, Provision, and Financing

## References

[1] Saikia AM Das J Barman P Bharali MD. Internet addiction and its relationships with depression, anxiety, and stress in urban adolescents of Kamrup District, Assam. J Fam Community Med. 2019;26(2):108. doi:10.4103/jfcm.JFCM_93_18 PMC6515762 31143082

[2] Cheng C Li AY. Internet addiction prevalence and quality of (real) life: a meta-analysis of 31 nations across seven world regions. Cyberpsychol Behav Soc Netw. 2014;17(12):755-760. doi:10.1089/cyber.2014.0317 25489876 PMC4267764

[3] Young KS. Internet addiction: the emergence of a new clinical disorder. Cyberpsychol Behav. 1998;1(3):237-244. doi:10.1089/CPB.1998.1.237

[4] Love T Laier C Brand M Hatch L Hajela R. Neuroscience of internet pornography addiction: a review and update. Behav Sci. 2015;5(3):388-433. doi:10.3390/bs5030388 26393658 PMC4600144

[5] Lin X Dong G Wang Q Du X. Abnormal gray matter and white matter volume in “Internet gaming addicts”. Addict Behav. 2015;40:137-143. doi:10.1016/j.addbeh.2014.09.010 25260201

[6] Liu X Gui Z Chen ZM et al. Global prevalence of internet addiction among university students: a systematic review and meta-analysis. Curr Opin Psychiatry. 2025;38(3):182-199. doi:10.1097/YCO.0000000000000994 40009750

[7] Ihekaike MM Shehu MY Makama M. Prevalence and associated factors of Internet addiction among clinical medical students of a Nigerian Private University. Int Neuropsychiatr Dis J. 2021;16(3):41-51. doi:10.9734/indj/2021/v16i330183

[8] Yang X Guo WJ Tao YJ , et al. A bidirectional association between internet addiction and depression: a large-sample longitudinal study among Chinese university students. J Affect Disord. 2022;299:416-424. doi:10.1016/j.jad.2021.12.013 34906641

[9] Lau JTF Walden DL Wu AMS Cheng KM Lau MCM Mo PKH . Bidirectional predictions between Internet addiction and probable depression among Chinese adolescents. J Behav Addict. 2018;7(3):633-643. doi:10.1556/2006.7.2018.87 30264608 PMC6426401

[10] Kuss DJ Kristensen AM Lopez-Fernandez O. Internet addictions outside of Europe: a systematic literature review. Comput Human Behav. 2021;115:106621. doi:10.1016/j.chb.2020.106621

[11] Endomba FT Demina A Meille V , et al. Prevalence of internet addiction in Africa: a systematic review and meta-analysis. J Behav Addict. 2022;11(3):739-753. doi:10.1556/2006.2022.00052 35984734 PMC9872524

[12] MellouliM ZammitN LimamM , et al. Prevalence and predictors of internet addiction among college students in sousse, Tunisia. J Res Health Sci. 2018;18(1):e00403.29445049

[13] Ogboghodo EO Omoregie EK Omoike E Omuemu VO. Knowledge of and attitude towards internet addiction among undergraduate students in University of benin, Edo State, Southern Nigeria. Niger J Health Sci. 2018;18(2):51. doi:10.4103/njhs.njhs_1_20

[14] Ahmed SK. How to choose a sampling technique and determine sample size for research: a simplified guide for researchers. Oral Oncology Reports. 2024;12:100662. doi:10.1016/j.oor.2024.100662

[15] Moon SJ Hwang JS Kim JY Shin AL Bae SM Kim JW. Psychometric properties of the internet addiction test: a systematic review and meta-analysis. Cyberpsychol Behav Soc Netw. 2018;21(8):473-484. doi:10.1089/cyber.2018.0154 30110200

[16] Kroenke K Spitzer RL Williams JB. The PHQ-9: validity of a Brief Depression Severity Measure. J Gen Intern Med. 2001; 16(9):606-613. doi:10.1046/j.1525-1497.2001.016009606.x 11556941 PMC1495268

[17] Ghasemi A Zahediasl S. Normality tests for statistical analysis: a guide for non-statisticians. Int J Endocrinol Metab. 2012;10(2):486-489. doi:10.5812/ijem.3505 23843808 PMC3693611

[18] Tabachnick B. Experimental Designs Using ANOVA. Thomson; 2007.

[19] Fallah M Mohajerani H Omidi A Ghaderi A. Targeting Internet addiction through Body Awareness Psychotherapy: the mediating role of executive functions and Emotion Regulation. Brain Behav. 2025;15(9):e70846. doi:10.1002/brb3.70846 PMC12405664 40898753

[20] Kheradmand A Amirlatifi E. Smartphone addiction and its associated factors among Tehran University students. BJPsych Open. 2022;8(Suppl 1):S40. doi:10.1192/bjo.2022.165

[21] Tateno M Teo AR Ukai W , et al. Internet addiction, smartphone addiction, and hikikomori trait in Japanese young adult: social isolation and social network. Front Psychiatry. 2019; 10:455. doi:10.3389/fpsyt.2019.00455 31354537 PMC6635695

[22] Yin KT Hj Yahaya A Sangryeol C Maakip I Voo P Maalip H. Smartphone usage, smartphone addiction, Internet Addiction and Nomophobia In University Malaysia Sabah (UMS). Southeast Asia Psychology Journal. 2019;7:1-12.

[23] Rathi M Guha P Neogi R. Internet addiction in adolescents: role of family, personality and comorbid psychopathology in school children in Eastern India. Indian J Psychiatry. 2022; 64(4):408-414. doi:10.4103/indianjpsychiatry.indianjpsychiatry_783_21 36060726 PMC9435623

[24] Ojedokun . Internet access and usage by students of the University of Botswana. Afr J Libr Arch Inf Sci. 2001;11(2):97-107.

[25] Bisen S Deshpande Y. Prevalence, predictors, psychological correlates of internet addiction among college students in India: a comprehensive study. Anatol J Psychiatr. 2020;21:1. doi:10.5455/apd.47328

[26] Sserunkuuma J Kaggwa MM Muwanguzi M , et al. Problematic use of the internet, smartphones, and social media among medical students and relationship with depression: an exploratory study. PLoS One. 2023;18:e0286424. doi:10.1371/journal.pone.02864245 May. PMC10218731 37235547

[27] Zenebe Y Kunno K Mekonnen M , et al. Prevalence and associated factors of internet addiction among undergraduate university students in Ethiopia: a community university-based cross-sectional study. BMC Psychol. 2021;9(1):4. doi:10.1186/s40359-020-00508-z 33407920 PMC7789664

[28] Niemz K Griffiths M Banyard P. Prevalence of pathological Internet use among university students and correlations with self-esteem, the General Health Questionnaire (GHQ), and disinhibition. Cyberpsychol Behav. 2005;8(6):562-570. doi:10.1089/cpb.2005.8.562 16332167

[29] Kuss D Griffiths M Binder J. 525 – Online on campus: internet addiction and personality in English university students. Eur Psychiatry. 2013;28:28(S1). doi:10.1016/s0924-93381375828-0

[30] Younes F Halawi G Jabbour H , et al. Internet addiction and relationships with insomnia, anxiety, depression, stress and self-esteem in university students: a cross-sectional designed study. PLoS One. 2016;11(9):e0161126. doi:10.1371/JOURNAL.PONE.0161126 PMC5019372 27618306

[31] Kutty RM Mahmood NHN Masrom M , et al. The influence of Internet Addiction and time spent on the Internet towards social isolation among university students in Malaysia. Asian Soc Sci. 2022;18(10):32. doi:10.5539/ass.v18n10p32

[32] Acharya S Adhikari L Khadka S Paudel S Kaphle M. Internet addiction and its associated factors among undergraduate students in Kathmandu, Nepal. J Addict. 2023;2023:1-9. doi:10.1155/2023/8782527 PMC10118885 37091192

[33] X M K GP Felix JWA . Prevalence and associated factors of Internet addiction among college students using smartphone in Tamil Nadu: a cross-sectional study. Int J Community Med Public Health. 2021;8(9):4525. doi:10.18203/2394-6040.ijcmph20213563

[34] Lin MP Ko HC Wu JY. The role of positive/negative outcome expectancy and refusal self-efficacy of internet use on internet addiction among college students in Taiwan. Cyberpsychol Behav. 2008;11(4):451-457. doi:10.1089/cpb.2007.0121 18721094

[35] Lin YJ Hsiao RC Liu TL Yen CF. Bidirectional relationships of psychiatric symptoms with internet addiction in college students: a prospective study. J Formos Med Assoc. 2020;119(6): 1093-1100. doi:10.1016/j.jfma.2019.10.006 31653577

[36] Zhao L Li X Yang Q , et al. The longitudinal association between internet addiction and depressive and anxiety symptoms among Chinese adolescents before and during the COVID-19 pandemic. Front Public Health. 2022;10:1096660. doi:10.3389/fpubh.2022.1096660 36743184 PMC9889652

