# Supplementary material for: Prevalence of Internet Addiction and Its Associated Factors Among Undergraduate Students at the University of Botswana: A Cross-Sectional Study
Source: Inquiry. 2026 Mar 24;63:00469580261432826. doi: 10.1177/00469580261432826 (PMC13013993; doi:10.1177/00469580261432826)
Supplement: sj-docx-1-inq-10.1177_00469580261432826 – Supplemental material for Prevalence of Internet Addiction and Its Associated Factors Among Undergraduate Students at the University of Botswana: A Cross-Sectional Study [file sj-docx-1-inq-10.1177_00469580261432826.docx]

STROBE Statement—Checklist of items that should be included in reports of ***cross-sectional studies***

**Study Title: Prevalence of Internet Addiction and Its Associated Factors among Undergraduate Students at the University of XXXX: A Cross-Sectional Study**

|  | Item No | Recommendation | Page No |
| --- | --- | --- | --- |
| **Title and abstract** | 1 | (*a*) Indicate the study’s design with a commonly used term in the title or the abstract | 1. Title |
|  |  | (*b*) Provide in the abstract an informative and balanced summary of what was done and what was found | 1. Abstract |
| Introduction | | | |
| Background/rationale | 2 | Explain the scientific background and rationale for the investigation being reported | 2. Introduction |
| Objectives | 3 | State specific objectives, including any prespecified hypotheses | 2. Introduction |
| Methods | | | |
| Study design | 4 | Present key elements of study design early in the paper | 4. (Methods, study design) |
| Setting | 5 | Describe the setting, locations, and relevant dates, including periods of recruitment, exposure, follow-up, and data collection | 4. (Methods, study setting) |
| Participants | 6 | (*a*) Give the eligibility criteria, and the sources and methods of selection of participants | 5. (Methods, Study Population and Inclusion Criteria) |
| Variables | 7 | Clearly define all outcomes, exposures, predictors, potential confounders, and effect modifiers. Give diagnostic criteria, if applicable | 6-7. (Methods, Sampling Technique, Recruitment Procedure) |
| Data sources/ measurement | 8* | For each variable of interest, give sources of data and details of methods of assessment (measurement). Describe comparability of assessment methods if there is more than one group | 7. (Methods, Measurement instruments |
| Bias | 9 | Describe any efforts to address potential sources of bias | 5. (Methods, Sample Size Determination)  7. (Methods, Measurement instruments) |
| Study size | 10 | Explain how the study size was arrived at | 5. (Methods, Sample Size Determination) |
| Quantitative variables | 11 | Explain how quantitative variables were handled in the analyses. If applicable, describe which groupings were chosen and why | 5. (Methods, Study Population and Inclusion Criteria) |
| Statistical methods | 12* | (*a*) Describe all statistical methods, including those used to control for confounding | 8. (Methods, Data analysis) |
|  |  | (*b*) Describe any methods used to examine subgroups and interactions | 8. (Methods, Data analysis) |
|  |  | (*c*) Explain how missing data were addressed | 8. (Methods, Data analysis) |
|  |  | (*d*) If applicable, describe analytical methods taking account of sampling strategy | 8. (Methods, Data analysis) |
|  |  | (*e*) Describe any sensitivity analyses | 8. (Methods, Data analysis) |
| Results | | | |
| Participants | 13* | (a) Report numbers of individuals at each stage of study—eg numbers potentially eligible, examined for eligibility, confirmed eligible, included in the study, completing follow-up, and analysed | 8. (Results) |
|  |  | (b) Give reasons for non-participation at each stage | 8. (Results) |
|  |  | (c) Consider use of a flow diagram | 8. (Results) |
| Descriptive data | 14* | (a) Give characteristics of study participants (eg demographic, clinical, social) and information on exposures and potential confounders | 8. (Results) |
|  |  | (b) Indicate number of participants with missing data for each variable of interest | 9. (Results) |
| Outcome data | 15* | Report numbers of outcome events or summary measures | 9. (Results) |
| Main results | 16* | (*a*) Give unadjusted estimates and, if applicable, confounder-adjusted estimates and their precision (eg, 95% confidence interval). Make clear which confounders were adjusted for and why they were included | 9. (Results) |
|  |  | (*b*) Report category boundaries when continuous variables were categorized | 9. (Results) |
|  |  | (*c*) If relevant, consider translating estimates of relative risk into absolute risk for a meaningful time period | 9. (Results) |
| Other analyses | 17 | Report other analyses done—eg analyses of subgroups and interactions, and sensitivity analyses | 9. (Results) |
| Discussion | | | |
| Key results | 18 | Summarise key results with reference to study objectives | 10. (Discussion) |
| Limitations | 19 | Discuss limitations of the study, taking into account sources of potential bias or imprecision. Discuss both direction and magnitude of any potential bias | 13. Discussion: (Strengths and limitations) |
| Interpretation | 20 | Give a cautious overall interpretation of results considering objectives, limitations, multiplicity of analyses, results from similar studies, and other relevant evidence | 12. Discussion |
| Generalisability | 21 | Discuss the generalisability (external validity) of the study results | 13. Discussion |
| Other information | | | |
| Funding | 22 | Give the source of funding and the role of the funders for the present study and, if applicable, for the original study on which the present article is based | 15. Funding statement |

*Give information separately for exposed and unexposed groups.

**Note:** An Explanation and Elaboration article discusses each checklist item and gives methodological background and published examples of transparent reporting. The STROBE checklist is best used in conjunction with this article (freely available on the Web sites of PLoS Medicine at http://www.plosmedicine.org/, Annals of Internal Medicine at http://www.annals.org/, and Epidemiology at http://www.epidem.com/). Information on the STROBE Initiative is available at [www.strobe-statement.org](http://www.strobe-statement.org).

**Item 12: Statistical methods**

(a) Statistical methods and control for confounding

Descriptive statistics were used to summarize the study variables, with continuous variables presented as means and standard deviations and categorical variables as frequencies and percentages. Inferential analyses were conducted using parametric methods due to the sufficiently large sample size. A single-step multiple linear regression analysis was performed to identify predictors of internet addiction. Variables included in the regression model were selected a priori based on evidence from the literature. Potential confounding was controlled for by simultaneously entering all selected independent variables into the multivariable regression model, allowing estimation of adjusted associations between predictors and the outcome. Statistical significance was set at p < 0.05.

(b) Subgroups and interactions

No formal subgroup analyses or interaction terms were examined in this study. The analysis focused on identifying overall predictors of internet addiction in the study population.

(c) Missing data

There were no missing data in the dataset; therefore, no imputation or other missing data handling methods were required.

(d) Analytical methods accounting for sampling strategy

No special analytical techniques were required to account for the sampling strategy, as the data were analysed as a single cross-sectional sample without weighting, clustering, or stratification.

(e) Sensitivity analyses

No sensitivity analyses were conducted.

**Item 13: Participants:**

(a) Numbers at each stage of the study
A total of 500 undergraduate students were recruited and participated in the study. All students who were approached met the eligibility criteria and were included in the analysis. All 500 participants completed the questionnaire and were included in the final statistical analyses.

(b) Reasons for non-participation
There were no refusals or exclusions after eligibility assessment, and no participants withdrew from the study. Consequently, there was no loss to follow-up.

(c) Flow diagram
Given that all eligible participants were included and analysed without attrition, a flow diagram was not considered necessary. However, a flow diagram may be provided for transparency if required by the journal.

**Item 14: Descriptive data**

(a) Characteristics of study participants
The study included 500 undergraduate students with a mean age of 21.42 years (SD = 4.36; range: 18–26 years). Females constituted 52.2% of the sample. A majority of participants (64.8%) reported high depression scores. Anxiety levels varied, with 56% classified as having normal anxiety, 34% mild anxiety, 9.4% moderate anxiety, and 0.6% severe anxiety. Substance use was reported by 32% of participants.

(b) Missing data
There were no missing data for any variables of interest; all analyses were conducted using complete cases (n = 500).

**Item 15: Outcome data**

Regarding internet use characteristics, smartphones were the most commonly used device (43.1%), followed by laptops (31.3%), desktop computers (12.5%), tablets (9.7%), and other devices such as smart TVs and smartwatches (3.4%). Social media use was the most frequently reported reason for internet use (91.8%), followed by academic purposes (89.2%). Other reported uses included listening to music (62.2%), watching movies (54.0%), downloading content (42.4%), online gaming (40.4%), pornography use (40.4%), forex trading (10.2%), and other purposes (12.8%).

Daily internet screen time varied, with 33.8% of participants spending less than 4 hours per day online, 46.2% spending between 5 and 8 hours, and 20.0% spending more than 8 hours per day.

Variables such as age, gender, depression, anxiety, substance use, device type, and screen time were considered potential confounders based on prior literature and were included in the regression model.

**Item 16: Main Results**

(a) Unadjusted and adjusted estimates
Multiple linear regression analysis was conducted to identify predictors of internet addiction. After adjustment for selected confounders informed by the literature (including age, gender, depression, anxiety, substance use, and hours spent on the internet), depression (B = 0.26; *p* < 0.001), anxiety (B = 0.12; *p* = 0.02), and number of hours spent on the internet (B = 0.33; *p* < 0.001) were significantly associated with internet addiction. The final model explained 30% of the variance in internet addiction scores (R² = 0.30), F(7, 492) = 29.6, *p* < 0.01. Unadjusted estimates are presented in Table 2 alongside adjusted coefficients.

(b) Category boundaries for categorized variables
Daily internet use was categorized as <4 hours, 5–8 hours, and >8 hours per day. Anxiety severity was categorized as normal, mild, moderate, and severe based on standard scale cut-off scores. Internet addiction severity was categorized as none, mild, moderate, and severe according to established scoring guidelines.

(c) Translation to absolute risk
Translation of relative estimates into absolute risk was not applicable, as the outcome was analysed as a continuous internet addiction score rather than a time-dependent or risk-based outcome.
